# HOW DO LONELY OLDER PEOPLE TALK ABOUT LONELINESS? PRELIMINARY ANALYSIS OF THE BBC LONELINESS EXPERIMENT

**DOI:** 10.1093/geroni/igac059.615

**Published:** 2022-12-20

**Authors:** Christina Victor, Manuela Barreto, Pamela Qualter

**Affiliations:** Brunel University London, London, England, United Kingdom; University of Exeter, Exeter, England, United Kingdom; University of Manchester, Manchester, England, United Kingdom

## Abstract

Three types of loneliness, social, emotional and existential, are identified in research, policy and practice. Do these categories reflect the language used by older adults to describe their experiences of loneliness? We use data from the 2018 BBC Loneliness Experiment and focus upon lonely adults aged 60 and older, living in the UK and with a maximum score of 9 on the UCLA loneliness scale. 1619 participants meet these criteria, 1480 provided a response to the question ‘’What does loneliness mean to you?" Participants ages ranged from 60-94; 90% aged 60-74 and 38% male. Free text answers ranged from 1-189 words, included both subjective (feeling alone) or objective (being alone) words and described social (no one to talk to), emotional (lack of closeness) and existential (lack of purpose) loneliness. Lonely older adults ‘talk’ about the three different types of loneliness singly or in combinations when explaining what loneliness means to them. We conclude that:- (a) existential loneliness merits more attention as it is less prominent in research compared with other types of loneliness and (b) lonely older adults describe different types of loneliness in the same answer.

